# OmniBio: an easy-to-use web app for kinetic growth parameter calculation from microplate reader data

**DOI:** 10.1093/bioadv/vbag074

**Published:** 2026-03-09

**Authors:** Sebastian Dehnhardt-Amengual, Catalina Ardiles, Ignacio Guarda, Isidora Achiardi-Letelier, Luis F Larrondo, Wladimir Mardones

**Affiliations:** Millennium Institute for Integrative Biology (iBio), Santiago, 8331150, Chile; Millennium Institute for Integrative Biology (iBio), Santiago, 8331150, Chile; Millennium Institute for Integrative Biology (iBio), Santiago, 8331150, Chile; Millennium Institute for Integrative Biology (iBio), Santiago, 8331150, Chile; Millennium Institute for Integrative Biology (iBio), Santiago, 8331150, Chile; Faculty of Biological Sciences, Pontificia Universidad Católica de Chile, Santiago, 8331150, Chile; Millennium Institute for Integrative Biology (iBio), Santiago, 8331150, Chile

## Abstract

OmniBio is a user-friendly web application designed to streamline the analysis of microbial growth curves from microplate reader data, eliminating the need for coding skills or cumbersome file preprocessing. Built in R, using the gcplyr package and Shiny framework, OmniBio supports data outputs from most commercial plate readers (i.e. Gen5 and iControl software), handling multiple sheets within a single file. Users can upload raw data along with experimental metadata to the platform, compute kinetic parameters such as maximum growth rate, lag time, ODmax, and area under the curve, and promptly visualize the results in real-time. The app produces plots for each parameter and a global heatmap analysis for comparing microbial performance across strains and culture media. Importantly, all the primary and processed data can be readily downloaded in a summarized Excel report. In contrast to existing tools, OmniBio optimizes the analysis process by automating repetitive computations and offering an intuitive, step-by-step workflow that does not require bioinformatics training. OmniBio is freely accessible at https://sdehnhardt.shinyapps.io/OmniBio_beta2/, and can be modified from open-source code on GitHub (https://github.com/sidehnhardt/OmniBio). The tool enables researchers to conduct high-throughput kinetic analysis in an efficient and user-friendly manner, optimizing both time and resource utilization.

## 1 Introduction

Bacterial growth curve analysis has been a fundamental strategy for understanding the physiological characteristics of microorganisms through the determination of their kinetic parameters (Fernández-Martínez *et al.* 2024). Typically, for this purpose, different kinetic parameters are compared across various culture media to determine phenotypic characteristics using a wide range of available software (e.g. GrowthRates or Microbial Lag Calculator) and R packages (e.g. GrowthCurver) (Hall *et al.* 2014, Sprouffske and Wagner 2016). Nevertheless, the use of R packages or other bioinformatics tools requires some skillsets or training, which complicates their widespread use by all lab members. With the advent of robotics in laboratory procedures, the need to process extensive data generated by this type of equipment has arisen, normally compromising significant analysis time. In this context, bioinformatics pipelines represent a viable alternative for automating repetitive tasks. Considering this, our goal is to use the gcplyr R package (Blazanin 2024), adapting it to handle multiple microplate readings of Optical Density in various culture media. Although the package already simplifies these tasks, we surmise that a user interface (UI) could provide a more efficient and real-time way to run the code in the backend while visualizing the results of the calculations. In this context, several user interface (UI)-based applications have been developed to facilitate growth curve analysis, including Parsley, QurvE, Welly, Dashing Growth Curves, and AUDIT (Blazanin 2024). These tools offer varying degrees of automation, visualization, and accessibility, and are valuable for specific analytical workflows. However, many UI-based applications implement custom or simplified analytical pipelines and do not expose the full flexibility of R-based frameworks. To our knowledge, none of these tools provides a graphical interface that directly implements a complete gcplyr-style analytical workflow, including data reshaping, blank correction, smoothing (which is beneficial not only for noisy data but also when density values are close to zero), derivative-based kinetic parameter estimation, and standardized visualization. With this in mind, we developed OmniBio, which can read and analyze data from software of any commercial microplate reader, handling several sheets within a single Excel file. In this platform, users can visualize the results of calculations in real-time and evaluate the growth kinetics in each well of a microplate. Also available is a tab that stores the results for fast comparisons between culture media and a tab with a Heatmap analysis that summarizes each of the parameters across experimental sets, permitting global comparative analyses of microbial behaviors. Additionally, all the calculations performed by OmniBio can be downloaded in a summarized Excel file. This tool is freely available at https://sdehnhardt.shinyapps.io/OmniBio_beta2/.

## 2 Workflow and features

**Figure 1:**
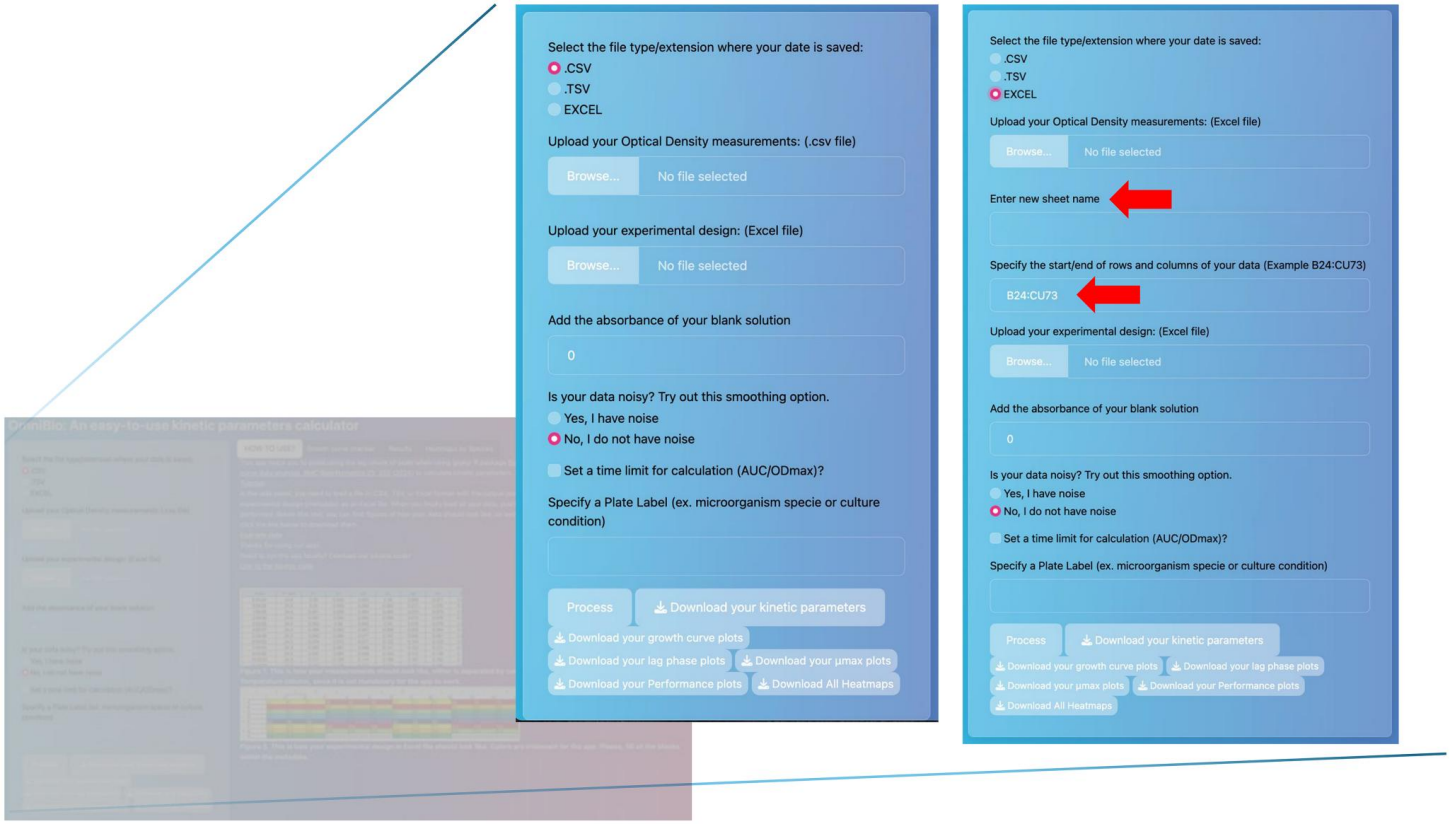
Snapshots of the different arguments for OmniBio analysis. The red arrow indicates the two fields that must be specified after selecting the file type. Note that this option appears only when the file type is selected in the first step.

One of the advantages of OmniBio is that, as hinted earlier, it does not require previous modification of the plate reader data output file. All calculations to determine the kinetic parameters are based on the code provided by the R package gcplyr (Blazanin 2024). For example, the Lag time calculation is calculated using the lag_time function, which defines lag time as the intersection between the x-axis and a tangent line drawn at the point of maximum per-capita growth rate. This tangent is projected backward to the minimum y-value, as described in the official gcplyr documentation. To start the analysis, the user must select the type of input file to be uploaded (CSV, TSV, or Excel format) as well as the metadata file (experimental design) used to identify the different samples in the assay. Once these are selected, the *sheet name* and the *start/end rows and columns* containing the optical density measurement data are enabled. This allows OmniBio to identify the location of the data within the sheet (Fig. 1).

Independent of the file type, a browsing list shows the available data sheets, allowing the user to perform multiple calculations of growth curve parameters using the same workbook. Note that the sheet name displayed corresponds to the one specified in the workbook. After selecting the sheet to be calculated, the user is required to enter a new name for the sheet, allowing the program to add it to the results file (it is allowed to enter letters and numbers in this box). The purpose of this new name is to serve as an identifier for the calculation results and to assign a clever reminder of each sheet analysis. This is useful if you are working with multiple culture media to understand the change in kinetic parameters for a particular microorganism.

Furthermore, it is necessary to upload another file corresponding to the “metadata of the experiment,” which is independent of the well plate used for the experiment. This metadata serves as an identifier for the different samples (i.e. strains, isolates, media composition/condition) across the plate, allowing the program to assign a name to each well and perform further calculations for the kinetic parameters. Finally, the platform requires adding the “plate label” and selecting the “Process” button. It does not matter which label is specified, since this only allows you to add the name to the final Excel results.

After entering all the above requirements, the web-based program starts to perform its calculations. Depending on the file size and the number of analyzed wells, processing takes a few seconds to nearly one minute to complete. To verify that the calculations were performed correctly, please navigate to the “Growth curve checker” tab and expect to see the plotted curves. Another way to check if the processing is finished is to observe the emergence of an informative pop-up, or to see if the download buttons are selectable.

OmniBio has various tabs to visualize the calculations and the values of the different kinetic parameters. Each of these tabs provides a brief description of the parameter, including its fundamentals. Additionally, OmniBio allows the use of blank values to subtract initial OD measurements, enabling more accurate calculation of parameters such as the area under the curve across different experiments. To get the post-processing results in Excel, the user must select “Download Kinetic Parameters”. Note that OmniBio can store the results of each sheet’s calculations, allowing the user to review and compare previous results with those from other sheets (e.g. comparing culture media).

**Figure 2:**
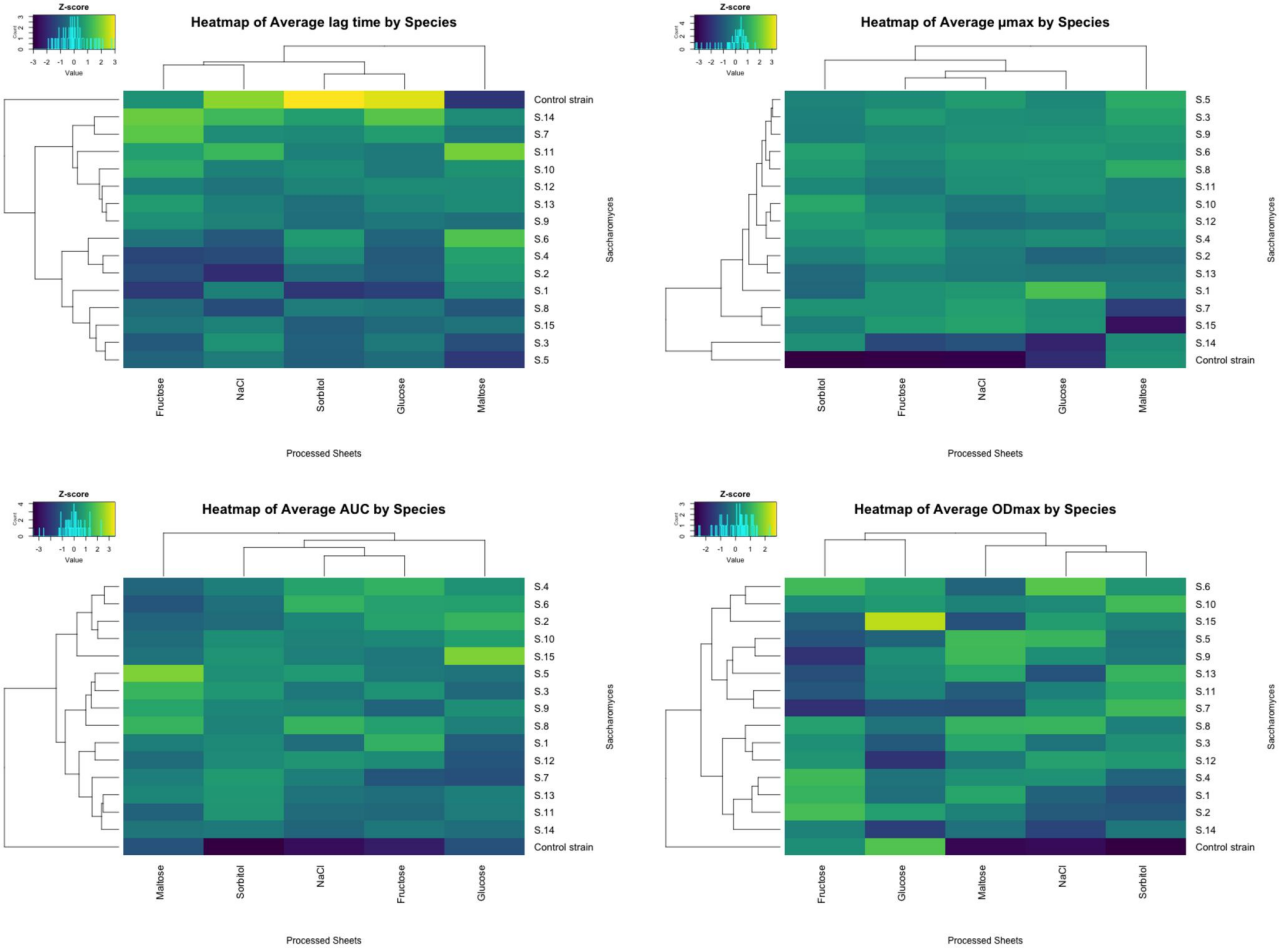
Heatmaps rendered by OmniBio with a multiple-sheet output file. In the Figure are shown four heatmaps, each corresponding to one of the kinetic parameters. The brighter color shows the higher Z-score value.

One of the functional analyses for high-throughput data analysis is a global comparison across strains and media cultures. For this, the heatmap provides a comprehensive alternative for intuitive data analysis (Cremonini 2024). The OmniBio platform can analyze the calculated data and generate a heatmap analysis for all kinetic parameters calculated. For this, the code compiles all the data generated across multiple sheets in a file, saves it in a temporary object, and scales the data (z-score) to visualize it in a heatmap with a dendrogram for columns and rows, using the R function “heatmap.2” of gplots library (Fig. 2). Indeed, in the provided example of the supplementary files, one can easily compare 16 yeast strains across 5 growth conditions, such as different carbon sources and osmotic stresses. The data were obtained from phenotyping assays of *Saccharomyces* genus strains, in which growth was monitored at 25 °C for 48 h using a Cytation 3 plate reader. The output figure permits comparing key growth parameters such as ODmax, lag time, µmax, and AUC. The heatmaps of the example data show that the control strain exhibits marked differences compared with the other strains, as evidenced by its separation into a distinct cluster (Fig. 2). Similarly, maltose emerges as a condition that induces differential behavior compared with the other culture conditions tested.

For more detailed instructions on how to use OmniBio, go to the supplemental material and watch the example video (Video S1). This material also includes sample data file (Table S2) with the respective metadata examples (Table S1).

OmniBio was developed entirely in the R language using RStudio and Shiny App programming logic. Furthermore, the complete code was published on the shinyapps.io server, accessible through the link: https://sdehnhardt.shinyapps.io/OmniBio_beta2/. The open-source code is fully available on GitHub (https://github.com/sidehnhardt/OmniBio).

## 3 Concluding remarks and limitations

OmniBio is a fast and user-friendly program for analyzing high-throughput data retrieved from practically all microplate reader software platforms. Furthermore, the app can handle the data workbook exactly as it is exported from the software, avoiding any data pre-processing or modifications that could lead to undesired errors. Although the software was originally developed for microbial growth curve analysis, it can also be applied to other types of response curves, such as luminescence signals from reporter assays, using the AUC parameter as a measure of induction. Future extensions of this app could include calculating diauxic shifts and compiling all the results obtained into one Excel file. Finally, due to the open-source nature of OmniBio, customization and improvement of the functionality of the software could be implemented.

## Supplementary Material

vbag074_Supplementary_Data

## Data Availability

The data underlying this article are available in GitHub at https://github.com/sidehnhardt/OmniBio.
